# Immune Modulatory Activities of Arginyl-Fructose (AF) and AF-Enriched Natural Products in In-Vitro and In-Vivo Animal Models

**DOI:** 10.3390/molecules26082251

**Published:** 2021-04-13

**Authors:** Jin-A Yu, Jung-Yun Lee, Tae Yang Kim, Hanna Kang, Su-Young Lee, Haimanot Mitiku, Joonheum Park, Young Hwan Lee, Hung-Bae Chang, Byung Ha Lee, Kichoon Lee, Emmanouil Apostolidis, Young-In Kwon

**Affiliations:** 1 Department of Food and Nutrition, Hannam University, Daejeon 34054, Korea; yja5546@daum.net (J.-A.Y.); seembeeks@hanmail.net (J.-Y.L.); xodid5606@naver.com (T.Y.K.); hanna9506@hanmail.net (H.K.); dltndud1221@naver.com (S.-Y.L.); mitikuhaimanot9@gmail.com (H.M.); 2 Institute of Functional Foods, EVERIT Co. Ltd., Daejeon 63010, Korea; jhpark@everit.kr (J.P.); sodamae2009@naver.com (Y.H.L.); 3 Department of Bio Quality Control, Korea Bio Polytechnic, Chungnam 32943, Korea; hbchang@kopo.ac.kr; 4 Department of Molecular Genetics and Center for Applied Plant Science, Ohio State University, Columbus, OH 43210, USA; leebyungha81@gmail.com; 5 Department of Animal Sciences, The Ohio State University, Columbus, OH 43210, USA; lee.2626@osu.edu; 6 Department of Chemistry and Food Science, Framingham State University, Framingham, MA 01701, USA

**Keywords:** ginseng, Amadori rearrangement compounds, adaptive immune, maillard reaction, arginyl-fructose

## Abstract

The immune system plays an important role in maintaining body homeostasis. Recent studies on the immune-enhancing effects of ginseng saponins have revealed more diverse mechanisms of action. Maillard reaction that occurs during the manufacturing processes of red ginseng produces a large amount of Amadori rearrangement compounds (ARCs), such as arginyl-fructose (AF). The antioxidant and anti-hyperglycemic effects of AF have been reported. However, the possible immune enhancing effects of non-saponin ginseng compounds, such as AF, have not been investigated. In this study the effects of AF and AF-enriched natural product (Ginofos, GF) on proliferation of normal mouse splenocytes were evaluated in vitro and male BALB/c mice models. The proliferation of splenocytes treated with mitogens (concanavalin A, lipopolysaccharide) were further increased by addition of AF (*p* < 0.01) or GF (*p* < 0.01), in a dose dependent manner. After the 10 days of oral administration of compounds, changes in weights of spleen and thymus, serum immunoglobulin, and expression of cytokines were measured as biomarkers of immune-enhancing potential in male BALB/c mice model. The AF or GF treated groups had higher weights of the thymus (0.94 ± 0.25 and 0.86 ± 0.18, *p* < 0.05, respectively) than that of cyclophosphamide treated group (0.59 ± 0.18). This result indicates that AF or AF-enriched extract (GF) increased humoral immunity against CY-induced immunosuppression. In addition, immunoglobulin contents and expression of cytokines including IgM (*p* < 0.01), IgG (*p* < 0.05), IL-2 (*p* < 0.01), IL-4 (*p* < 0.01), IL-6 (*p* < 0.01), and IFN-γ (*p* < 0.05) were also significantly increased by supplementation of AF or GF. These results indicate that AF has immune enhancing effects by activation of adaptive immunity via increase of expression of immunoglobulins and cytokines such as IgM, IgG, IL-2, IL-4, IL-6 and thereby proliferating the weight of thymus. Our findings provide a pharmacological rationale for AF-enriched natural products such as ginseng and red ginseng that can possibly have immune-enhancement potential and should be further evaluated.

## 1. Introduction

Immunity is a self-defense system that exists in the body, and is the process by which the human body recognizes, removes and metabolizes various foreign invaders, such as toxins and living organisms. It prevents the damage from external stimuli and the invasion of pathogenic microorganisms, but it can also damage own and healthy tissues, by triggering non-specific inflammatory reactions [1]. When immune system is compromised, immunomodulation is initiated through our immune system, which maintains homeostasis within the immune system.

Immunity is separated into innate immunity and adaptive immunity. The adaptive immunity, that is investigated in this study is mediated by B cells and T cells, and such conversion of the immune response is performed by the reaction of parameters associated with the activation of T cells [2,3,4]. There are two types of T cell activation, called T helper type 1 (T_H_1) and T helper type 2 (T_H_2), depending on the type of cytokine that is secreted when the T cell CD4 + receptor is activated by an immune response [5]. Types of Cytokines secreted from T_H_1 include IL-1, IL-2, IL-12, IL-15, IL-18, IFN-γ, and TNF-α, which are cytotoxic as inflammatory cytokines. Cytokine types secreted from T_H_2, which has a cell-mediated immune response, include IL-4, IL-5, IL-6, IL-10, IL-13, etc. and their purpose is to activate B cells [6,7,8]. It has been reported that it regulates the associated humoral immune response and protects against extracellular pathogens and has the ability to suppress cell immunosuppression and T_H_1 cell activity [9,10]. Based on the above, the immunosuppressive and immune enhancing effect of bioactive substances from natural products have been studied for their potential regulation and changes in the above-mentioned immune function.

Ginseng is an old medicinal plant that has been used for medicinal purposes in Asia for centuries. In particular, the immune-enhancing effects of ginseng and its saponin compounds have been confirmed in human clinical trials [11,12]. More specifically, studies on the immune-enhancing effects of heat processed ginseng (red ginseng) saponins have been performed, and more diverse mechanisms of action are being studied in recent years [13,14]. During steaming and drying, the various chemical components of Korean red ginseng undergo a nonenzymatic browning reaction, otherwise known as a Maillard reaction resulting to the production of Maillard reaction products (MRPs). Among these components, Amadori compounds, a result of non-enzymatic glycation end-products are generated by the Maillard reaction. In the early stage of Maillard reaction, a large amount of Amadori rearrangemnet compounds (ARCs) such as arginyl-fructose (AF) are formed through amadori rearrangement of arginine with glucose during the red ginseng manufacturing process. It has been reported that the content of AF in commercially available ginseng products is around 2% (dry weight basis) [15]. Many researchers are now conducting research to determine the potential health benefits of AF resulting from the heat-processing of ginseng, such as antidiabetic [16,17] and antioxidant [18] effects. However, studies on the immune enhancing activity of non-saponin based compounds present in red ginseng, such as AF are very limited [19].

Recently, the Korean Ginseng Research Institute reported an adaptive immune enhancing effect of AFG, one of ARCs in red ginseng [19]. However, very limited information on the immune enhancing effect of other ARCs is available in processed ginseng. Therefore, the aims of this manuscript are (1) to measure the bioactive biomarker AF content in the extract of heat-processed ginseng (Ginofos, GF) product, and (2) to investigate the adaptive immunity improving effects of GF and AF, which is an indicator of GF and physiological activity. To evaluate the immune enhancing effect of AF and GF, the cell proliferation activities of AF and GF were measured using immune related cells such as splenocytes and thymus ex-vivo. Furthermore, to prove the ability and efficacy of adaptive immunity of these samples, immunoglobulins and cytokines expression were investigated in-vivo animal models.

## 2. Results

### 2.1. HPLC Analysis of AF

**Figure 1:**
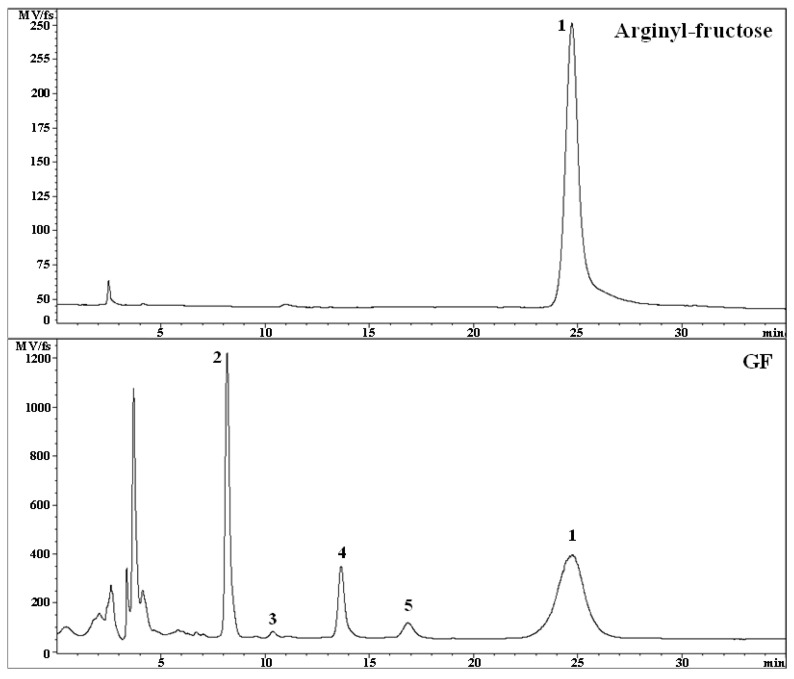
HPLC Analysis of GF components using ELSD (**1**: Arginyl-fructose (AF), **2**: Fructose, **3**: Glucose, **4**: Sucrose, **5**: Maltose).

The HPLC profile of the GF sample used for the evaluation test of the improving effect of immune function was investigated. The results of measuring the AF content in GF were shown in Table 1 and Figure 1. The AF content in the GF sample was 3.61% (Table 1), which is the highest content compared to other components present in the GF sample, such as mono- and disaccharides in the tested sample.

From these results, it was found that AF is the main bioactive compound in GF. Additionally, these results indicate that GF has a relatively high AF content compared with the previous research results reported by Yoo et al. [15], which reported that the AF content in commercially available ginseng products was around 1–2%. Therefore, it was considered that AF could play a role of key factor for the immunity function of GF.

### 2.2. Effects of AF and GF on Spleen Cell Proliferation

AF-enriched samples (GF) at a dose of 1, 10, 100 μg/mL, and its bioactive biomarker AF at a dose of 0.35, 3.5, 35 μg/mL were added into spleen cells then incubated for 48 h. Negative control were prepared as described in the materials and methods, but without the addition of GF or AF samples. Positive control group were treated with concanavalin A (Con A) or lipopolysaccharide (LPS) to stimulate T cell or B cell proliferation, respectively. The group treated with Con A or LPS have higher absorbance than those of the group cultured only spleen cells without the addition of mitogen (data not shown).

**Figure 2:**
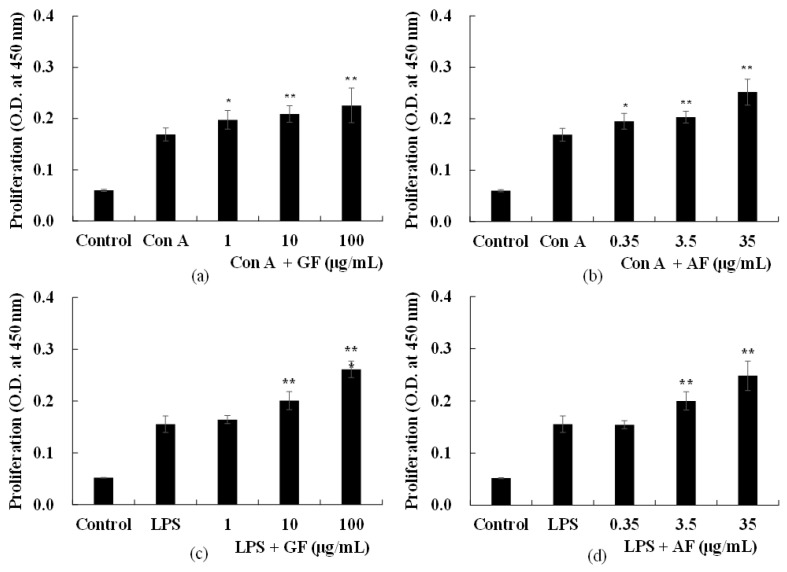
Splenocyte proliferation induced by Con A and LPS of Ginofos extract (GF), Arginyl-fructose (AF) ex-vivo. Mouse Splenocytes were stimulated with Con A (15 μg/mL), LPS (500 μg/mL) in the presence of various concentration of GF, AF for 48 h. Con A group (GF: (**a**), AF: (**b**), LPS group (GF: (**c**), AF: (**d**) was cultured with Con A alone. The splenocyte proliferation was assessed by MTT. The results are expressed as the mean ± S.D of different experiments and all experiments were done in triplicate. Statistical significances from Con A group were determined by Student’s *t*-test (* *p* < 0.05, ** *p* < 0.01).

To understand the effect of GF and AF on Con A-induced T cell response, Con A, GF (1, 10, and 100 μg/mL) or AF (0.35, 3.5, 35 μg/mL) were added into culture media then cultured for 48 h. The proliferation of splenocytes treated with Con A were further increased by addition of GF (*p* < 0.05 and *p* < 0.01) or AF (below *p* < 0.05), significantly, in dose dependent manners (Figure 2a,b). Furthermore, to evaluate the effect of GF and AF on B cell proliferation induced by LPS, GF (1, 10, and 100 μg/mL) or AF (0.35, 3.5, 35 μg/mL) were added into culture media with LPS. After incubating for 48 h, absorbance was measured by spectrometer at 450 nm. GF and AF treated groups showed significantly increase of cell proliferation in a dose dependent manner (below *p* < 0.05), compared to the absorbance of the control group treated with only LPS, similar with the test results under Con A treatment (Figure 2c,d).

Spleen cell proliferation induced by LPS increased by GF and AF treatments, suggesting that GF and its biomarker AF may exhibit an immune-promoting effect to affect the humoral immune response. In addition, in the case of AF treatment group showed a spleen cell proliferation effect in a three times lower concentration compared to the GF treatment group. These results demonstrate that the humoral immune enhancing effect of GF is due to AF.

Taken together, increased spleen cell proliferation induced by GF and AF with Con A suggests that GF and AF are effective in cellular immune response by promoting T cell proliferation.

### 2.3. Effects of AF and GF on Spleen Antibody Production

#### 2.3.1. Body Weight and Immune-Related Organ Weight

Cyclophosphamide (CY) is used as an alkylating agent in cells to bind to DNA and interfere with the normal functioning of cells to manage inflammation and combat cancer cells. In addition, when applied to lymphocytes, it exhibits immunosuppressant activity or has serious side effects, and is metabolized by alkylating agents in the liver, and is cross-connected with DNA to form new DNA strands. It has been reported that alkylation inhibits DNA synthesis from T cells and B cells, thus impairing the functions of T and B cells and reducing immunity [20,21,22]. Therefore, this study was conducted to investigate how immunosuppression was improved by the combined administration of this immunosuppressant and GF/AF.

GF or AF was administered orally daily for 10 days. CY was administrated intraperitoneally as a single dose of 100 mg/kg on day 5 before sacrifice. As shown in Table 2, there were differences in the weight of liver and kidney due to CY administration, and between the GF and AF treated groups. The weight of both thymus and spleen relevant for immunity was significantly reduced in all groups administered CY, causing immune decline due to CY (Table 2). The weight of spleen in CY control decreased by 48%, but it was around 43% and 42% in GF and AF-treated groups with a non-significant difference, suggesting recovery tendency. The thymus weight of CY control group decreased significantly (*p* < 0.05) (77%) compared to normal control. GF or AF with CY administration groups, significantly increased (by 46% and 60%, respectively), compared to the thymus weight of the CY control group (*p* < 0.05). These results indicate that GF and AF could assist towards recovering the weight of the thymus and spleen, possibly by protecting and enhancing CY-induced damage of the immune system. As a result of the above experiments, GF and AF could increase thymus weight, and improve the immune function by activating the humoral immune response inhibited by CY.

#### 2.3.2. Blood Immunoglobulin (IgM and IgG) Contents

Table 3 shows the results of measuring serum proteins that play a major role in humoral immunity (adaptive immunity).

By showing that the values of all immunoglobulins in the normal control group (untreated group) were higher than those in the CY control group, it was possible to know that the values of immunoglobulin in the blood were indicators for the improvement and enhancement of immunity.

It was found that the values of two immunoglobulins were significantly improved. More specifically, blood IgM level was significantly increased in the GF (430.37 ± 55.92) and AF (430.37 ± 78.83) groups compared to the CY control (230.37 ± 61.20) group. Although not as much as IgM, IgG also showed a significantly increased pattern (Table 3). Based on the results in Table 3, immunoglobulin induced by GF and AF indicated its involvement in possible strengthening immune function.

#### 2.3.3. Measurement of Hepatotoxicity and Cytokine Expression in Spleen Cells

**Figure 3:**
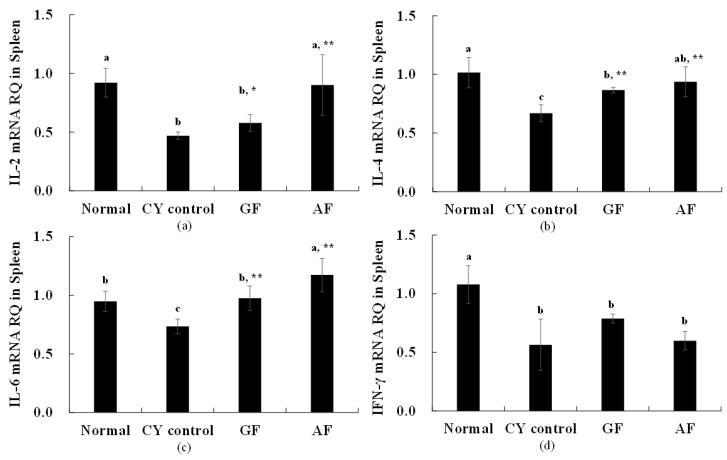
RT-real time PCR quantitative analysis of cytokine mRNA expression in the Spleen of *Balb/c* mice. For real time PCR, we used SYBR green mix with cytokine gene-specific primers (**a**): IL-2, (**b**): IL-4, (**c**): IL-6, (**d**): IFN-γ). The level of protein expression was normalized with GAPDH. Each vale is expressed as mean ± S.D. and is representative of at least three separate experiments. ^a–c^ Different letters indicate statistically significant differences between groups one-way ANOVA followed by Duncan’s test of *p* < 0.05. The results are expressed as the mean ± S.D of 5 animals per group. Statistical significances from CY control group were determined by Student’s *t*-test (* *p* < 0.05, ** *p* < 0.01).

Cytokines are produced by several immune cells and have been reported to have the potential to affect activation, growth, differentiation, etc. of some immune cells. Cytokine proteins are not only involved in innate and adaptive immune responses, but also play important roles in the process of immune cell maturation. In this study, using mice model we monitored the immune system was decreased by CY administration, and evaluated whether or not this decrease was improved by administration of GF and AF, through the possible positive effect on cytokine expression level (Table 4 and Figure 3).

TNF-α cytokines, which mediate and regulate innate immunity, are the major mediators of the acute inflammatory response of Gram-negative bacteria and other infectious agents and have been reported to cause severe infections and complications. TNF-α is also secreted by antigen-stimulated T lymphocytes, NK cells, and mast cells, but activated macrophages are the main producing cells. In Table 4 we can observe the effect of GF and AF administration on TNF-α, which was not significantly improved compared to the CY control.

Meanwhile, to evaluate the liver toxicity of AF and GF, alanine aspartate aminotransferases (AST) and alanine aminotransferases (ALT) as biochemical indicators showing hepatotoxicity in blood were measured. Under normal conditions, these diagnostic enzymes have low enzymatic activity, but can cause lesions such as tissue necrosis due to damage to hepatocytes resulting from chemical poisoning, bacterial infections, tumors, or hypoxia. When induced, their activity in the serum is increased. In this study, as a result of analyzing AST and ALT values vs. normal control group to confirm whether hepatotoxicity was induced after administration of GF and AF substances, the CY control (408.52 ± 126.03) group resulted to a significantly increased AST value. Based on this observation, the CY administration was used to understand if GF and AF administration could possibly improve AST values. Our results suggest that GF and AF administration results to reduction of hepatotoxicity, as suggested by the reduced AST levels (Table 4).

When IL-2 expression was evaluated, we observed that CY control resulted to a significantly lower expression, compared to the normal control (Figure 3). GF and AF administration significantly increased the expression of IL-2 compared to the CY control group (Figure 3). Similar observations occurred with IL-4, where the normal control group was 1.02 ± 0.13, and the CY control resulted to reduced IL-4 levels (0.67 ± 0.07). It was confirmed that the expression of IL-4 was significantly increased in the group treated with GF (0.87 ± 0.02) and AF (0.94 ± 0.13) when compared to the CY control group. In the case of IL-6 expression level, the expression level was 0.95 ± 0.09 in the normal control group, and significantly lowered in the CY control (0.74 ± 0.06) group. It was confirmed that the expression of IL-6 was significantly increased in the group treated with GF (0.96 ± 0.10) and AF (1.17 ± 0.14) when compared to the CY control group. Finally, when INF-γ expression level was evaluated, the normal control group was 1.08 ± 0.16, the CY control (0.56 ± 0.22) group was significantly lower, and the GF and AF-treated group yielded to no significant difference in INF-γ expression when compared to the CY control group.

Promoting production, IL-4 is involved in the production of IgE antibodies and the main stimulus for T_H_2 cell development, and IL-6 stimulates the growth of differentiated B cells and produces anti-inflammatory cytokines [5,23].

As described above, it was found that the expression of IL-2, IL-4 and IL-6which are cytokines that mediate adaptive immunity, is increased and immune cells are promoted. Through this study, the improvement of cytokine expression related to adaptive immunity in spleen cells with the administration of GF and AF to animals with weakened immunity is shown to be effective in improving immunity associated with the promotion of immune cells.

## 3. Discussion

In this study, GF containing around 3.6% of AF and AF alone were used to demonstrate the effect of improving immunity through evaluation of the ability and efficacy of adaptive immunity and measurement of cytokine expression.

Initially, GF and AF treatments reduced the negative effects of Con A and LPS treatments by measuring the proliferative capacity of spleen cells (Figure 2). The observed stimulation occurred in a dose-dependent manner and suggests that AF can be bioactive components within GF for the observed bioactivity. Then CY, which is an alkylating agent, was used to suppress the rejection displayed after organ transplantation and treating malignant tumors, but also normal cells due to its well-defined non-selective toxicity. CY is also toxic, and similar side effects as anemia, leukocyte and thrombocytopenia have been reported [22]. As a result of proceeding to oral administration of GF and AF for 10 days to an animal model in which humoral immune function was reduced by CY treatment, the intake of food and drinking water and the type of behavior between the normal control, CY control, and sample treatment groups were monitored. No significant differences were observed between all groups. The effects of CY control, GF + CY and AF+CY on the thymus and the spleen were monitored. In the case of spleen, significant difference was observed between the treatments and the CY control (Table 2). Furthermore, the thymus showed increased growth rates with GF or AF administration, when compared to the CY control (Table 2). Previous study showed that arginyl-fructosyl-glucose (AFG)-enriched ginseng extracts restored the weights of the spleen and thymus, which were reduced by CY [19]. Therefore, these results indicate that a little difference in composition of sugar moiety between AF and AFG may increase humoral immunity against CY-induced immunosuppression in animal models.

When the liver biomarkers (AST and ALT) were evaluated we observed that GF and AF administration improved the AST levels, which were impaired due to CY administration. All other biomarkers remained similar to the control, suggesting that there is no hepatotoxicity due to the ingestion of GF and AF (Table 4).

When the content of the cytokine TNF-α was measured, which mediates and regulates innate immunity, no statistical difference was observed between all groups (Table 4). To better understand the immunity effect of the tested samples, the expression of IL-2, IL-4, IL-6, and INF-γ was measured (Figure 3).

IL-2, IL-4, IL-6, and INF-γ are cytokines that mediate and regulate adaptive immunity [24]. mRNA was extracted from the spleen cells of animals that were administered CY, GF+CY and AF+CY. Expression of IL-4 and IL-6 were increased with both GF and AF treatment (compared to CY control), expression of IL-2 was increased only with AF treatment (compared to CY control) and no effect was observed in the expression of INF-γ (Figure 3).

Serum proteins that play a major role in humoral (adaptive) immunity with antibody function are classified into IgA, IgD, IgE, IgG, and IgM [1]. The immunoglobulin IgG measured in this study binds to phagocytes as most antibodies in the serum and is involved in pathogen phagocytosis and destruction, and IgM is first secreted upon initial antigen contact. Immunoglobulin that promotes antigen neutralization and aggregation reaction is involved in complement activation. Compared to the CY control group, IgM (GF 430.37 ± 55.92, AF 430.37 ± 78.83), IgG (GF 395.30 ± 25.03, AF 398.33 ± 62.71) treated groups were statistically improved immunoglobulin secretion (Table 3).

## 4. Materials and Methods

### 4.1. Materials

Arginyl-fructose (AF) standard was purchased from Proteinworks Co. Ltd. (Daejeon, Korea). Ginofos (GF), a natural product mixture, was donated by PRIMINE Co. Ltd. (Daejeon, Korea) which made a variety of medicinal plants in Korea. GF contains heat processed red ginseng extract (above 30 wt%). These plants were extracted using hot water (90 °C) for 24 h, and evaporated by Liquefied extractor (PRIMINE Co. Ltd., Daejeon, Korea) to yield a powder of Ginofos. BALB/c mice and feeds (corn starch, casein, vitamin mix, mineral mix, calcium phosphate, and sodium chloride) for animal trials were purchased from Raon Bio (Yonginsi, Korea). Lipopolysaccharide (LPS), concanavalin A (Con A), Earle’s Balanced Salt Solution (EBSS), DEAE-dextran, agar, 2-mercaptoethanol, and cyclophosphamide were purchased from Sigma-Aldrich Co. (St. Louis, MO, USA). In addition, RPMI 1640 medium, fetal bovine serum (FBS), and HEPES buffer were purchased from Gibco BRL (Grand Island, NY, USA). SRBCs were purchased from South Pacific Sera Ltd. (Timaru, New Zealand). Unless noted, all other chemicals were purchased from Sigma-Aldrich.

### 4.2. HPLC Analysis of AF

The analysis was performed by a method of Joo and others [15] using high-performance anion-exchange chromatography with electrochemical detector (ICS-2500, Dionex Co., Sunnyvale, CA, USA). The chromatographic separation was carried out with a CarboPac PA1 anion-exchange column (250 mm × 4 mm, Dionex Co.) and a CarboPac PA1 guard column (50 mm × 4 mm, Dionex Co.). The column temperature was 30 °C. The elution was performed with water (A) and 400 mM sodium hydroxide (B) (10:90, *v*/*v*) using binary gradient elution. The flow rate was 0.7 mL/min and injection volume were 20 μL. The AF was detected by electrochemical detector equipped with a gold working electrode and Ag/AgCl reference electrode operating quadruple potential waveform.

### 4.3. Cell Proliferation Effect of AF and GF

After sacrificing 7-week-old male BALB/c mice using cervical dislocation, the spleen was aseptically removed and stored into RPMI 1646 medium (10% FBS, 1% penicillin, 15 mM HEPES buffer, 2 mM). The sample was washed with L-glutamine (50 nM 2-mercaptoethanol), weakly shredded with large-sized needles on sterile slides to from single cells, and centrifuge with conditions of 3000× *g* and 4 °C for 10 min. Cell pellets were resuspended in ACK lysis buffer for 2 min to remove erythrocytes, centrifuged again in RPMI 1646 medium twice, and then cell number was counted. The number of cells was adjusted to 5 × 10^5^ cells/well 160 μL, dispensed into a 96-well plate, and then used for measuring the cell proliferation capacity (ability) of the spleen. Diluted the spleen cell suspension to 5 × 10^5^ cells/well 180 μL, and added 20 μL each of LPS (500 μg/mL) and Con A (15 μg/mL) in 96-well plate. GF and AF were treated one by one and used in the positive control group (AF). After treating GF (1, 10, 100 μg/mL) and AF (0.35, 3.5, 35 μg/mL) in wells by 10 μL, incubate for 48 h under the conditions of 37 °C and 5% CO_2_ to give an MTT indicator. After treating 20 μL each individually at 37 °C for 2 h with 5% CO_2_, after removing the culture solution, 200 μL 1× PBS washing, DMSO 100 μL put and measuring the absorbance at 570 nm to measure the spleen cell proliferation ability evaluated [19].

### 4.4. Animal Study

All animal procedures were approved by Institutional Animal Care and Use Committee (IACUC) of the Hannam University (Approval number: HNU2020-002). Six-week-old male BALB/c mice were purchased from Orient Bio Co. (Yongin, Korea) and fed a solid diet (Samyang Diet Co., Seoul, Korea) for one week. The mice were housed in a ventilated room at 25 ± 2 °C with 50 ± 7% relative humidity and under an alternating 12 h light/dark cycle, and all air in the breeding space (SPF zone) uses air that has been filtered through a HEPA filter. Animals were divided into four groups:

I: Normal control (fed saline only) − Normal
II: Negative control (100 mg/kg of cyclophosphamide) − CY Control
III: 100 mg/kg Ginofos extract (GF)
IV: 35 mg/kg arginyl-fructose (AF)

GF (100 mg/kg) and AF (35 mg/kg) were administered to 6-week-old BALB/*c* mice twice daily (9 am and 5 pm) for 10 days, using a gavage needle, respectively. In the normal control group (Normal) and the immunocompromised group (CY Control), the same amount of distilled water was orally administered daily for 10 days. In order to induce a decrease in immunity with CY (cyclophosphamide), 100 mg/kg of CY was intraperitoneally administered on the day when the test substance was started and 5 days after the administration.

### 4.5. Evaluation of the Ability to Improve Immunity

#### 4.5.1. Changes in Weight of Body, Thymus, Spleen and Liver

Mice were weighed from the test substance administration start date (day 0) and CY administration date (day 5) to the day of autopsy during the test period. The test animals were anesthetized with CO_2_ gas and the spleen, liver and thymus were removed and weighed.

#### 4.5.2. Measurement of Antibody-Producing Ability in Mice Immunocompromised to CY

Using a mouse immunoglobulin G (IgG) and immunoglobulin M (IgM) ELISA kit (K3231083 and K3231088, Koma Biotech CO., Ltd., Seoul, Korea), the IgG and IgM contents in the plasma of animals (BALB/c mice) collected after oral administration of GF and AF for 10 days were measured. After washing the 96-well plate with 300 μL washing solution (1× PBS, 0.05% Tween-20, pH 7.4), removed the residual buffer, put 100 μL of the samples in plasma, standard, and blank wells, reacted at 25 °C for 1 h, and then washed wells with 300 μL of washing solution (1X PBS, 0.05% Tween-20, pH 7.4) 4 times repeatedly, added 100 μL of detection antibody (IgG or IgM) and reacted at 25 °C for 1 h. After repeatedly washed wells 4 times with 300 μL of washing solution, added 100 μL of TMB solution for reaction at 25 °C for 10 min. After that, 100 μL of stop solution (0.18 M, H_2_SO_4_) was added, the absorbance was measured at 450 nm, and the antibody content in the blood was measured [20].

#### 4.5.3. TNF-α and Expression of Cytokines

Using the mouse TNF-α ELISA kit (BMS607-3, Thermo Fisher Scientific Korea, Co., Ltd., Seoul, Korea), the amount of TNF-α in animal (BALB/c mice) in plasma after oral administration of GF and AF for 10 days was measured. After washing the 96-well plate with 400 μL of washing solution (1X PBS, 0.05% Tween^TM^-20), removed the residual buffer, added 50 μL + biotin-conjugate 50 μL solution in each of plasma, standard, and blank wells, and incubated at 25 °C for 2 h. After that, washed wells with 400 μL of washing solution 6 times repeatedly, added 1× Streptavidin HRP solution 100 μL and reacted well plate at 25 °C for 1 h, then washed repeatedly with 400 μL of washing solution (1X PBS, 0.05% Tween^TM^ 20) six times, and added TMB solution 100 μL. The solution was added and subjected to a color reaction at 25 °C for 30 min, then stop solution 100 μL of 1 M phosphoric acid was added, the absorbance was measured at 450 nm, and the TNF-α content in the blood was measured.

After oral administration of GF and AF for 10 days, the intracellular cytokine expression level in the spleen excised from each mouse was measured using the real time PCR method. RNA was extracted from animal tissues using a TRI reagent solution, and then cDNA was synthesized using a high-capacity cDNA reverse transcription kit (K1622, Thermo Fisher Scientific Korea, Co., Ltd.). The primers listed below were used to measure the expression levels of adaptive immune-related cytokine (Interleukins; IL-2, IL-4, IL-6, and Interferon; IFN-γ) genes and GAPDH genes (Table 5). Using the QuantiTect SYBR Green PCR kit (A25741, applied biosystems by Thermo Fisher Scientific Korea, Co., Ltd.), which utilizes the characteristics of SYBR Green sandwiched between dsDNA together with oligomers synthesized, each gene was amplified. After confirming the amplification process by 7500-Real-Time PCR system, the degree of expression was quantified by comparing with the expression level of GAPDH as an internal control.

### 4.6. Analysis of Blood Biochemical Properties

AST (aspartate aminotransferase) and ALT (alanine aminotransferase) activities were measured to evaluate the level of liver damage and the effects of AF and GF administration on liver function. After oral administration of GF and AF for 10 days, using the Aspartate aminotransferase kit (Precedure No. 2920/2930, Stanbio Laboratory, L.P., Boerne, TX, USA), 100 μL of plasma of the collected from animals (BALB/c mice) was mixed with 1 mL of Aspartate aminotransferase enzyme solution, and then measured activities using an automatic blood analyzer (Shinging Sun A6, Dongsung CO., Ltd., Seoul, Korea).

### 4.7. Statistical Analysis

All data are presented as mean ± S.D. Statistical analyses were carried out using the statistical package SPSS 11 (Statistical Package for Social Science, SPSS Inc., Chicago, IL, USA) program and significance of each group was verified with the analysis of One-way ANOVA followed by Duncan’s test of *p* < 0.05. In addition, statistical significances in animal study were determined by Student’s *t*-test (* *p* < 0.05; ** *p* < 0.01; and *** *p* < 0.001).

## 5. Conclusions

GF containing around 3.6% AF and AF alone promote B and T cells in spleen cells. Some cytokines secreted from spleen cells (IL-2, IL-4 and IL-6) seem to be activated with the treatments, but no effect was observed on INF-γ and TNF-α. Additionally, we observed an increase in secreted blood immunoglobulins. No liver toxicity was observed with our treatments and in the case of AST, GF and AF treatments yielded to an improvement when compared to the CY control. This study provides a basic biochemical rationale for the further evaluation of the possible mechanism of action of GF and AF for an immunomodulation effect. To better understand the mechanism of action, in the future we would like to evaluate the effect of GF on more biological markers involved to immune response using different animal-stress treatments.

## Figures and Tables

**Table 1:** Arginyl-fructose (AF) and Saccharides Contents in Ginofos extract (GF).

	Ginofos Extract (%)
Arginyl-fructose	3.61 ± 0.18
Fructose	1.51 ± 0.11
Glucose	0.14 ± 0.02
Sucrose	0.49 ± 0.13
Maltose	0.30 ± 0.02

**Table 2:** Body weight and organ weight in male BALB/c mice.

	Normal Control	CY Control	CY + GF	CY + AF
Body weight (g)	21.76 ± 0.62 ^a^	19.79 ± 1.12 ^b^	17.74 ± 0.86 ^c^	18.52 ± 0.95 ^c^
Liver (mg/g)	41.94 ± 2.23 ^a^	42.89 ± 2.29 ^b^	40.00 ± 3.29 ^b^	38.29 ± 3.84 ^c^
Kidney (mg/g)	12.88 ± 0.60 ^a^	13.61 ± 0.65 ^b^	13.16 ± 1.16 ^b^	13.26 ± 1.21 ^b^
Thymus (mg/g)	2.61 ± 0.24 ^a^	0.59 ± 0.18 ^b^	0.86 ± 0.18 ^b,^*	0.94 ± 0.25 ^b,^*
Spleen (mg/g)	2.85 ± 0.08 ^a^	1.47 ± 0.22 ^b^	1.61 ± 0.04 ^b^	1.63 ± 0.08 ^b^

The results are expressed as the mean ± S.D of five animals per group. ^a–c^ Different letters indicate statistically significant differences between groups one-way ANOVA followed by Duncan’s test of *p* < 0.05. Statistical significances from CY control group were determined by Student’s *t*-test (* *p* < 0.05).

**Table 3:** Effect of GF and AF on the plasma IgG, IgM level in BALB/c mice immunosuppressed by cyclophosphamide.

	Normal Control	CY Control	CY + GF	CY + AF
IgM (μg/mL)	530.37 ± 44.91 ^a^	230.37 ± 61.20 ^b^	430.37 ± 55.92 ^a,^*	430.37 ± 78.83 ^a,^*
IgG (μg/mL)	618.03 ± 151.33 ^a^	216.52 ± 68.23 ^c^	395.03 ± 25.03 ^b,^*	398.33 ± 62.71 ^b,^*

The results are expressed as the mean ± S.D of 5 animals per group. ^a–c^ Different letters indicate statistically significant differences between groups one-way ANOVA followed by Duncan’s test of *p* < 0.05. Statistical significances from CY control group were determined by Student’s *t*-test (* *p* < 0.05).

**Table 4:** Plasma cytokine (TNF-α) and AST, ALT profile in male BALB/c mice after 10 day on the experimental immunology.

	Normal Control	CY Control	CY + GF	CY + AF
AST (mg/dL)	249.68 ± 60.72 ^b^	408.52 ± 126.03 ^a^	200.88 ± 68.88 ^b,^*	299.06 ± 101.20 ^ab^
ALT (mg/dL)	35.74 ± 7.55 ^a^	40.92 ± 14.04 ^a^	33.92 ± 14.04 ^a^	31.50 ± 4.47 ^a^
TNF-α (pg/dL)	111.90 ± 31.74 ^a^	91.38 ± 3.33 ^a^	87.06 ± 6.16 ^a^	100.52 ± 12.70 ^a^

The results are expressed as the mean ± S.D of five animals per group. ^a–c^ Different letters indicate statistically significant differences between groups one-way ANOVA followed by Duncan’s test of *p* < 0.05. Statistical significances from CY control group were determined by Student’s *t*-test (* *p* < 0.05).

**Table 5:** Primers for real-time quantitative PCR.

Genes	Primer Sequences	
	Forward (5′–3′)	Reverse (5′–3′)
GAPDH	CCACCCAGAAGACTGTGGATGGC	CATGTAGGCCATGAGGTCCACCAC
IL-2	TCCACCACAGTTGCTGACTC	CCTGCATCTAGAGGCTGTCC
IL-4	CGAAGAACACCACAGAGAGTGAGCT	GACTCATTCATGGTGCAGCTTATCG
IL-6	AGAGGAGACTTCACAGAGGA	ATCTCTCTGAAGGACTCTGG
IFN-γ	GCGTCATTGAATCACACCTG	TGAGCTCATTGAATGCTTGG

PCR: polymerase chain reaction, GAPDH: glyceraldehyde 3-phosphate dehydrogenase, IL: Interleukin, IFN: Interferon.

## Data Availability

The data will be available on request.

## Sample Availability

Samples of the compounds (Ginofos) are available from the authors.
